# Discrepancies in the evaluation of the safety of the human papillomavirus vaccine

**DOI:** 10.1590/0074-02760180063

**Published:** 2018-05-28

**Authors:** Jorge L Cervantes, Amy Hoanganh Doan

**Affiliations:** Texas Tech University Health Sciences Center, Paul L Foster School of Medicine, El Paso, TX, USA

**Keywords:** papillomavirus, vaccine, autoimmunity

## Abstract

Despite being more than ten years since its introduction, global acceptance to the human papillomavirus (HPV) vaccine is still low. The immunogenetic background of the host, and HPV antigen recognition, are important in natural HPV infection, and should be taken into account in the understanding of adverse autoimmune reactions by the HPV vaccine in certain groups. There is no doubt of the benefit of vaccines in the reduction of the incidence of infectious diseases, and in the case of HPV, the prevention of persistent infection that would lead to cervical cancer. Side-effects, however, should be closely monitored and reported without any bias, to ensure that the benefits of vaccines outweigh the risks of adverse reactions. In this article we bring the attention on certain adverse effects of the vaccine against HPV that have not been well studied as they are not well defined. We also compare the different approaches on HPV vaccine policies regarding its adverse reactions in countries like Japan and Colombia, vs. the recommendations issued by the WHO.

*The vaccine against the human papillomavirus (HPV)* - HPV infection is the most common sexually transmitted viral infection. Several risk factors for HPV infection have been identified, and include genetic predisposition, immune status, co-infection with other sexually transmitted diseases, and smoking. Persistent infection with this virus is associated with squamous carcinoma of the cervix, oropharynx, anus, genitalia (vulva, vagina and penis), head, and neck. Approximately 90% of HPV infections resolve spontaneously through the immune system. Persistent infection with high-risk types 16 and 18 contributes to 20% and 50% of cervical cancers, respectively (Skeate et al. 2016). It is thus, not surprising that the first HPV vaccines were directed toward these genotypes (Muñoz et al. 2008). There are 13 other high risk genotypes, including 31, 33, 35, 39, 45, 51, 52, 56, 58, 59, 68, 73 and 82 (Skeate et al. 2016), whose presence in certain populations of Latin America (Cervantes et al. 2003), could explain the discrepancy between the prevalence of 16/18 genotypes and the incidence of cervical cancer. A newer nonavalent vaccine has been approved by the Food and Drug Administration (FDA) to account for infections by seven high-risk HPV genotypes (16, 18, 31, 33, 45, 52, and 58) (Kirby 2015), as the HPV vaccine does not provide protection against other types than those included in the vaccine.

Current HPV vaccines are based on virus-like particles, and are composed of self-assembled pentamers of the larger protein of the L1 capsid. HPV vaccines are prophylactic and are not therapeutic. The goal of prophylactic HPV vaccination is to avoid persistent infections that will progress to an invasive carcinoma. HPV vaccination would not be appropriate to elicit an anti-cancer response, since the tumor cells do not express significant levels of L1 protein. For treatment of cancers originating from HPV there is immunotherapy, which focuses on generating a cellular immune response against antigens associated with cellular transformation (Skeate et al. 2016). The HPV vaccine does not modify the cellular immunity that is responsible for eliminating the infected cells, rather it induces the production of antibodies against the L1 protein in blood. The two main HPV vaccines, Gardasil (Merck) and Cervarix (GSK), were approved in 2006 and in 2009, respectively, so it is still difficult to predict its long-term efficacy.

*Rejection of the HPV vaccine* - Even though more than 10 years have passed since its introduction, the global acceptance of the HPV vaccine remains low. In Latin America, the immunization rate is lower than expected (Tabakman 2017). In developed countries such as Canada, the rejection of HPV vaccination is high in both the lowest and the highest economic levels (Remes et al. 2014).

Several countries in Latin America have shown a marked decrease in their immunization rates (Tabakman 2017). The initial recommendation of three intramuscular doses has now been reduced to two doses in a period of 6 to 24 months, in order to reduce costs and increase compliance (Handler et al. 2015). Adherence to the three doses of the vaccine has also been low in Latin American countries, such as Brazil, Mexico, and Argentina (Tabakman 2017). This occurs after complaints of various symptoms, including suicides in Colombian girls after receiving the vaccine (Tabakman 2017).

Japan, after four years since its introduction, suspended the recommendation to immunize against HPV in 2014 (Larson et al. 2014). The decision was made after reported cases of chronic pain and other symptoms. Despite the reviews claiming that these reported cases were not related to the vaccine, no agreement was reached between Japan and the World Health Organization (WHO). Japan has previously shown to have very stringent thresholds for risk acceptance. One example of such, occurred during the bovine spongiform encephalopathy crisis, where, Japan re-examined meat samples imported from the US regardless of the safety assurance from the United States Department of Agriculture (USDA) and ultimately decided to suspend the importation of meat from the United States.

*Adverse reactions to the HPV vaccine* - According to HPV vaccine manufacturers, the most common adverse reactions to Gardasil include pain, swelling, redness, stinging, bruising, bleeding at the injection site, and headache, fever, nausea, diarrhea, abdominal pain, and syncope (https://www.fda.gov/downloads/BiologicsBloodVaccines/Vaccines/ApprovedProducts/UCM111263.pdf). For Cervarix, local adverse reactions occurring in ≥ 20% of subjects are pain, redness, and swelling at the site of injection. The most common general adverse events in ≥ 20% of the subjects are fatigue, headache, myalgia, gastrointestinal symptoms, and arthralgia (FDA).

The most frequent reported symptoms of HPV vaccination are chronic pain with paresthesia, headaches, fatigue, and orthostatic intolerance (Martínez-Lavin 2015). Small series and isolated cases of complex regional pain syndrome (CRPS), postural orthostatic tachycardia syndrome (POTS), and fibromyalgia, have been reported after vaccination against HPV. These conditions are often difficult to diagnose, and show similar clinical characteristics (Goldenberg 2009).

Apparently, dysfunction of the sympathetic nervous system plays an important role in the pathogenesis of these syndromes (Martínez-Lavin 2015). Ninety three percent of affected subjects continue to have disabling symptoms for more than four years, unable to return to school or work (Tomljenovic et al. 2014, Martínez-Lavin et al. 2015). Other studies, nevertheless, have shown a lack of evidence of an association between HPV vaccine and CRPS (Weinbaum and Cano 2015) or fatigue (Donegan et al. 2013, Feiring et al. 2017).

It should be mentioned that chronic arthropathy has also been observed with other vaccines, such as the rubella vaccine.

*HPV vaccine safety* - The recommendation for the use of HPV vaccine by the WHO is based on efficacy and effectiveness data (WHO 2017a). The Global Advisory Committee for the Safety of Vaccines (GACVS) is an independent body composed of clinicians and expert scientists, who meet under the WHO to provide rigorous advice on the safety of vaccines of global importance. In its latest report, the committee has evaluated the risk of developing Guillain-Barré syndrome, concluding that the risk is no more than 1 case per 1 million vaccinated (WHO 2017b). The committee has also found no evidence of causality between the HPV vaccine and SDRC or STPO, and considers that there is still no evidence suggestive of a causal association for the various symptoms (including pain and motor dysfunction) after reviewing data obtained from surveillance in Japan. A review of the HPV vaccine safety by the Centers for Disease Control and Prevention (CDC) found no difference in side effects between vaccinated and unvaccinated individuals (Gee et al. 2016). In fact the CDC's Vaccine Adverse Event reporting System (VAERS) states that the HPV vaccine is very safe, and has not found any unexpected patterns in maternal or fetal outcomes (Moro et al. 2015).

These conclusions, however, are based on records that should be interpreted with care, especially when assessing cases with non-specific diagnosis, for which there is not a clear consensus on the diagnostic criteria (Goldenberg 2009) (Fig. 1).

**Fig. 1:**
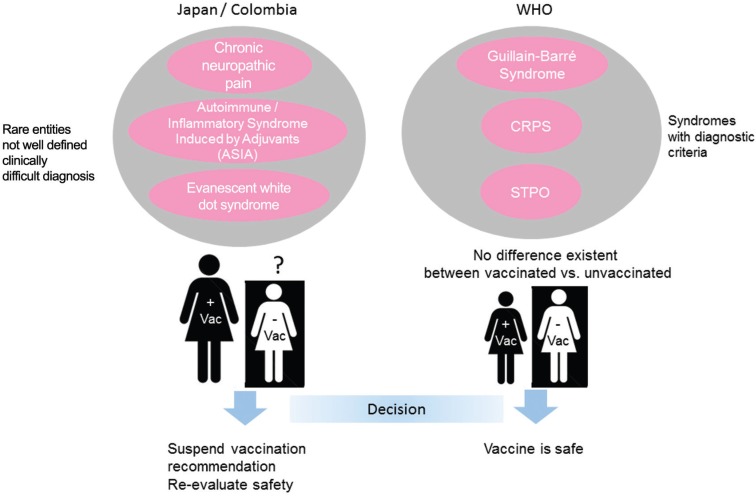
discrepancies in the evaluation of the human papillomavirus (HPV) vaccine. Authorities from countries such as Japan and Colombia, made the decision to suspend the vaccination recommendation and re-evaluate the vaccine, due to a significant number of cases of rare or clinically undefined complications after reception of the vaccine. The World Health Organization, on the other hand, evaluated the safety of the vaccine using more defined categories, and made its recommendations based on the efficacy and effectiveness of the vaccine.

Despite these pronouncements on the safety of HPV vaccine, regions in Colombia have reported a disproportionate number of neuropathic pain cases (with respect to the expected reactions declared by the pharmaceutical company producing Gardasil) (Sánchez-Gómez and Hernández-Flórez 2014).

It is important to note that the occurrence of demyelinating disease after vaccination, despite being low, is not negligible. This type of complication has been reported for multiple vaccines such as influenza, HPV, hepatitis A or B, rabies, measles, rubella, yellow fever, anthrax, meningococcus, and tetanus.

*Post-vaccination syndromes: beyond adjuvants* - The presence of viral nucleic acids can cause autoimmune phenomena (Jeffs et al. 2016). HPV vaccines, however, are composed of proteins as we previously described. An adjuvant is a substance commonly used in vaccines in order to increase the immune response against an antigen. The quadrivalent vaccine of Gardasil (Merck) contains a simple adjuvant of aluminum hydroxide (225 mg). The nonavalent vaccine contains twice the concentration of adjuvant (500 mg of Aluminum)(Gee et al. 2016). The Cervarix vaccine uses a proprietary adjuvant, 3-O-desacyl-4 monophosphoryl lipid A (AS04), which appears to be more potent (Handler et al. 2015).

The term “ASIA” (Autoimmune/inflammatory Syndrome Induced by Adjuvants) describes a spectrum of clinical conditions that share similar signs and symptoms, including somatoform and dysautonomic post-vaccination phenomena (Palmieri et al. 2017). With respect to the HPV vaccine, it has been estimated that the rate of ASIA syndrome is 3.6 cases per 100,000 doses of anti-HPV vaccine (95% CI 3.4-3.7). The most common clinical manifestations are pyrexia (58%), myalgia (27%) and arthralgia or arthritis (19%).

It would not be the first time that adjuvants in a vaccine cause an adverse reaction. The use of AS03 as an adjuvant in the Pandemix vaccine against the influenza virus was linked to the development of autoimmune narcolepsy.

A recent study, however, shows that aluminum present in the adjuvant does not play a role in cellular hypersensitivity (Poddighe et al. 2017). On the other hand, a review of the adverse effects in clinical trials comparing women who received the bivalent vaccine vs. those who received the aluminum placebo, showed an increase in deaths in the vaccinated group. This result cautions of a projected greater systemic adverse effects with the nonavalent vaccine. Furthermore, there is also a report of autoimmune thrombocytopenia with antiphospholipid antibodies after HPV vaccination (Bizjak et al. 2016).

Equally important, the absence of symptoms does not exclude the presence of an inflammatory phenomenon, with epipharingitis being found in all women examined after HPV vaccination in Japan (Hotta et al. 2017). This phenomenon is more severe if the patient has a predisposition to develop autoimmune diseases such as lupus (Soldevilla et al. 2012) (Fig. 2). Another reported post-HPV vaccination syndrome is called multiple evanescent white dot syndrome, a rare retinopathy of unknown origin (Ogino et al. 2014). The favorable response to immunosuppressive therapy suggests an autoimmune phenomenon, or at least an imbalance in immune function (Fig. 2).

**Fig. 2:**
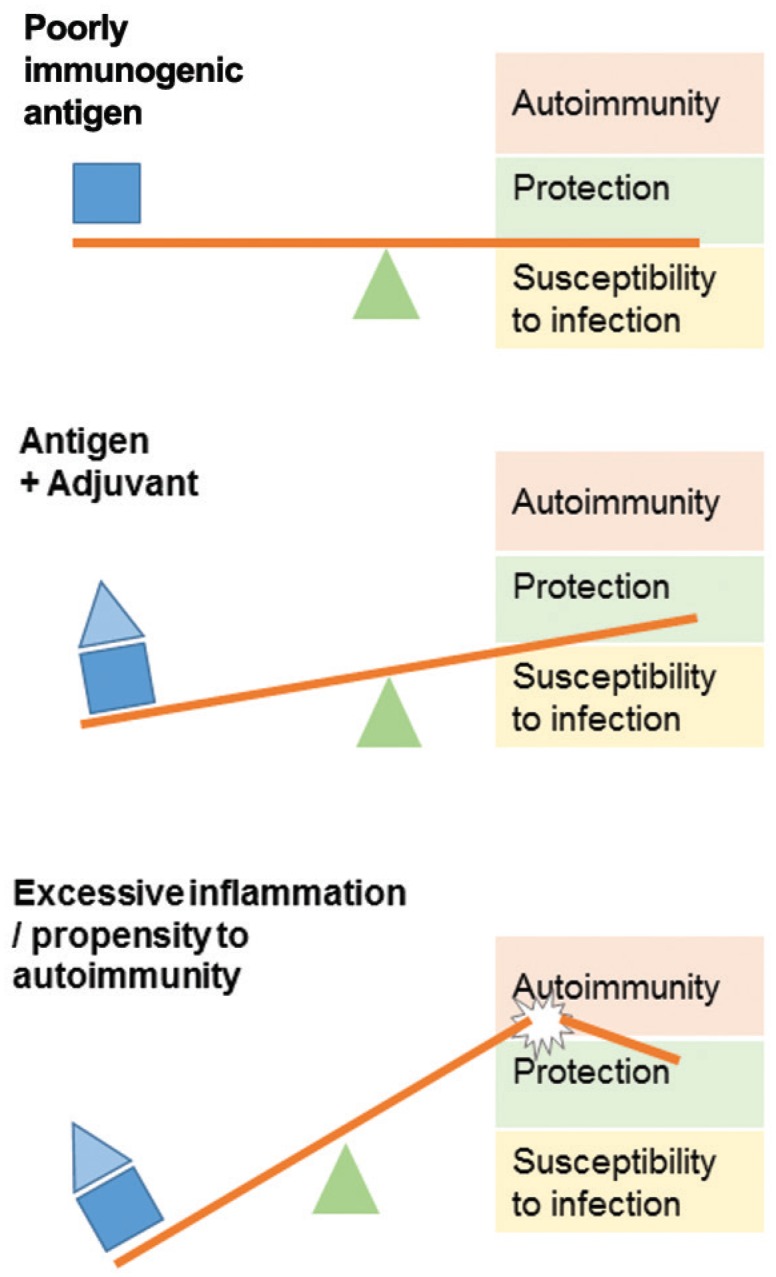
differences in the response to adjuvants. To overcome the obstacle of low immunogenicity of an antigen, protein-based vaccines often use adjuvants to obtain adequate levels of protection. In individuals with a certain predisposition to the development of autoimmunity, the excessive immune response can trigger autoimmune phenomena.

This evidence justifies the call made in countries such as Colombia for the identification of predictive markers to develop autoimmune diseases in the population to be vaccinated against HPV.

*In conclusion* - Apart from cultural and religious barriers, the adverse effects to HPV vaccines must be re-evaluated, since the initial clinical trials of the first vaccines were tested in different populations than women in Latin America or Japan. The role of the host immunogenetic background in HPV infection and the recognition of HPV antigens (Cervantes 2011) are important, and have been studied for decades. The benefit of vaccines is undoubtedly to reduce the incidence of infectious diseases, and in the case of HPV, prevent the development of persistent infections leading to cervical cancer. Even so, the side effects must be closely monitored, and reported without bias, to ensure that the benefits outweigh the risks.
